# Antibiotic Treatment Decrease the Fitness of Honeybee (*Apis mellifera*) Larvae

**DOI:** 10.3390/insects12040301

**Published:** 2021-03-30

**Authors:** Xinle Duan, Bi’an Zhao, Xin Jin, Xuefen Cheng, Shaokang Huang, Jianghong Li

**Affiliations:** 1 College of Animal Science (Bee Science), Fujian Agriculture and Forestry University, Fuzhou 350002, China; xinleduan@fafu.edu.cn (X.D.); zba.fafu@outlook.com (B.Z.); 1180744007@fafu.edu.cn (X.J.); 1190744002@fafu.edu.cn (X.C.); skhuang@fafu.edu.cn (S.H.); 2 Fujian Honey Bee Biology Observation Station, Ministry of Agriculture and Rural Affairs, Fuzhou 350002, China

**Keywords:** honeybee larvae, symbiotic bacteria, development, nutrient metabolism, immunity

## Abstract

**Simple Summary:**

To determine the biologic function of gut bacteria with no host specificity in honeybee larvae, honeybee larvae were treated with antibiotics for disrupting the gut bacteria. Then, the body weight, development time, and expression of nutrient metabolism genes and immune genes of honeybee larvae were investigated. The results demonstrated that the disruption of gut microbiota by antibiotics weakened the nutrient metabolism, decreased the body weight, extended the development process, and decrease the immune competence of honeybee larvae, indicating the vital roles of gut bacteria in bee larvae fitness.

**Abstract:**

Symbiotic bacteria could increase the nutrient provision, regulate the physiological state, and promote immunity in their insect host. Honeybee larvae harbor plenty of bacteria in their gut, but their functions are not well studied. To determine their effect on honeybee larvae, the 1-day-old larvae were grafted on to 24-well plates from the comb and artificially reared in the lab. They were treated with penicillin–streptomycin to remove the gut symbiotic bacteria. Then, the 5-day-old larvae and the newly emerged adults were weighted. The developmental periods to pupae and eclosion were investigated, respectively. The bacterial amount, expression of developmental regulation genes (*ecr* and *usp*), nutrient metabolism genes (*ilp*1, *ilp*2, *hex* 70*a*, *hex* 70*b*, *hex* 70*c*, and *hex* 110), and immunity genes (*apidaecin*, *abaecin*, *defensin-1*, and *hymenoptaecin*) were determined by qRT-PCR. The result showed that the antibiotics-treated larvae have significantly lower body weights in the 5-day-old larvae and the emerged bees. The expression of *ilp*2 and *hex* 70*c* in 5-day-old larvae was down-regulated. The *usp* was down-regulated in 5-day-old larvae, but increased in 7-day-old larvae, which disturbed the normal developmental process and caused the extension of eclosion. Moreover, antibiotics treatment significantly decreased the expression of *apidaecin* and *abaecin* in 5-day-old larvae, and *defensin-1* and *hymenoptaecin* in 7-day-old larvae, respectively. These results showed that antibiotics could weaken the nutrient metabolism, disturb the development process, and decrease the immune competence of honeybee larvae, indicating the vital roles of gut bacteria in bee larvae fitness, so the antibiotics should be avoided to control microbial disease in honeybee larvae.

## 1. Introduction

The honeybee is among the most economically important insects; it not only pollinates agricultural crops and wild plants, which account for EUR 153 billion per year worldwide, but also provide abundant bee products [1,2,3]. In recent years, the dramatic reductions in bee colonies, causing significant economic losses, were reported from all over the world [4,5,6,7,8]. The main reasons were diverse agrochemicals, parasites, viruses, changes of planting structure and distribution, or their interaction factors [5].

In animals, from humans to insects, the gut-dwelling microbial communities are indispensable for their host’s health [9,10]. The gut symbionts could shape the physiology, behavior, and fitness traits of the host. In honeybees, a distinctive community of bacterial species was found in the guts of honeybee workers [11]. Five core microbiotas (*Snodgrassella alvi*, *Gilliamella apicola*, *Lactobacillus Firm-4*, *Lactobacillus Firm-5*, and *Bifidobacterium*) and other four bacterial strains (*Frischella perrara*, *Bartonella apis*, *Parasaccharibacter apium*, and *Alpha* 2.1) constitute the main gut microbiota of honeybees [12]. The microbial profile was affected by the in-hive tasks of honeybees [13]. It was also influenced by many factors such as veterinary drugs, dietary supplements, antibiotics, and non-protein amino acids [14]. These gut microbiota could promote the food digestion, nutrition provision, and body weight of adult bees [15]. The nutrient level of an adult bee in a colony determines its labor division: nurse bees with a high level of nutrient state and foragers with a low level of nutrient state [16]. Low levels of the nutrient will lead to precocious foraging [17,18]. Adult bees with gut bacteria removed or disrupted by antibiotics have low nutrition levels, which increase the gustatory responses for food requirements [19]. These bees also have an earlier transition from a nurse bee to a forager, which indicates the gut bacteria can participate in the regulation of the honeybee behavioral development process [20].

Moreover, the microbiota could protect the hosts against pathogens either through indirect ways of increasing the host’s immunity or direct ways of the antagonist [21,22,23]. So, the host microbiota was considered to be an “extended immune phenotype” in addition to the host immune system itself [24]. Compared with the great achievements in the structure and function of gut microbiota in adult bees, the function of gut microbiota in larva bees has not been fully determined. The gut microbial communities in larva bees were simple and could be easily influenced by food and the environment [23]. Acetobacteraceae *Alpha 2.2* could live in as early as 1st and 2nd instar larval guts; *L. kunkeei* and *L.* Firm-5 were detected in later instars. These bacteria could contribute to larval immunity during the early stages of honeybee development [25]. The lactic acid bacteria (LAB) in food could significantly reduce the honeybee larvae infected by the pathogen of American foul brood [26]. Thus, gut bacteria play a positive role in honeybee larvae [27,28].

Honeybee larvae are sensitive to nutrient provision. Queen larvae feeding on royal jelly have a better nutrient state, shorter developmental period, heavier body weight, and longer life span, which are all opposite to the worker larvae with a low nutrient level [29]. Considering the comprehensive function of gut bacteria in adult bees in nutrient provision and the regulation of physiological states, behavior, and immunity, we hypothesize a similar role of gut bacteria in honeybee larvae. To verify this, antibiotics were used to disturb the symbiotic bacteria of honeybee larva guts in this study; then, the development parameters of larvae were investigated. Moreover, the expression levels of two development-regulation genes (*ecr* and *usp*), six nutrient metabolism-related genes (*ilp1, ilp2, hex 70a, hex 70b, hex 70c*, and *hex 110*), and four antimicrobial peptide genes (*apidaecin, abaecin, defensin*-1, and *hymenoptaecin*) were determined. The results showed that the disruption of gut bacteria by antibiotics weakened the nutrition metabolism, extended the development process, and inhibited host larva immunity, which verified the vital roles of gut bacteria in honeybee larvae and provide a theoretical basis for related research and application in the beekeeping industry.

## 2. Materials and Methods

### 2.1. Honeybee

The experimental honeybees were collected from the healthy colonies kept in the experimental apiary of the College of Animal Science (College of Bee Science), Fujian Agriculture and Forestry University (Fuzhou, China). The test larvae were obtained by the following method. Three normal egg-laying queens were separately confined on empty combs which were placed in a queen egg-laying controller for laying eggs for 8 h; then, the combs with new-laid eggs were moved to a separated place in the same colony by a queen excluder. Four days later, the comb with the 2-day-old larvae was swiftly transferred to the laboratory and kept in an incubator at 34 ± 1 °C.

### 2.2. Antibiotic Treatment of A. mellifera Larvae

The larvae were reared according to the method of Jensen et al. [30] with a few modifications. In detail, the 2-day-old larvae in the three combs kept in the incubator were randomly transferred into six 48-well tissue culture plates with 10 μL artificial diet in each well by using the Chinese grafting tool. Three plates were taken as the control group, and the left three plates were taken as the treatment group. The artificial diet formula referred to that in Vojvodic et al. [31]. These larvae were fed once per day based on the quantity, as described by Nie et al. [32]. The plates were kept in a dark incubator at 34 ± 1 °C, relative humanity 95 ± 2%. To avoid the negative influence of some antibiotic-resistant bacteria formed in the beekeeping industry by the use of antibiotics, such as tetracycline or tylosin, for controlling honeybee bacterial diseases, such as American Foulbrood Diseases (AFB) and European Foulbrood Diseases (EFB), the antibiotic combination, penicillin–streptomycin, which is rarely used in beekeeping, was used for inhibiting the bacteria in larvae. For the treatment group, larvae were fed a diet containing penicillin–streptomycin (1/50, *v*/*w*, Sangon Biotech Co., Ltd., Shanghai, China). The control group larvae were fed the normal diet without penicillin–streptomycin. To determine the effect of antibiotics on larva development, the time (hours) of pupation and eclosion in each group was recorded through observation from 6 h before the standard developmental time of pupation and emergence. The weight of new pupae and emerged bees was measured by an electronic balance (Sartorius, BS124S) individually for investigating the effect of gut bacteria on larva nutrition. Moreover, four 3-, 5-, and 7-day-old larvae, respectively, were randomly sampled from each culture plate; a total of 72 larvae were sampled from the treatment and control groups. These samples were immediately stored at −80 °C for RNA extraction.

### 2.3. RNA Isolation and cDNA Synthesis

Total RNA was extracted from the larvae stored in −80 °C using TRIzol^®^ Reagent (Invitrogen, Carlsbad, CA, USA), according to the bench protocol. The quality and concentration of the total RNA were determined by NanoDrop One (Thermo Fisher Scientific, Waltham, MA, USA); the qualified RNA samples were stored at −80 °C. cDNA was synthesized using the Hifair^®^ II 1st Strand cDNA Synthesis SuperMix for qPCR (gDNA digester plus) based on the protocol (Yeasen Biotech Co., Ltd., Shanghai, China). The qualified cDNA samples were stored at −20 °C.

### 2.4. Quantitative Real-Time PCR (qPCR)

The qPCR assay was performed to examine the quantity of gut bacteria using the universal bacteria primer (*unibac*), and the expression levels of two development regulation genes, *ecdysone receptor* (*ecr*) and *ultraspiracle protein* (*usp*); six nutrient metabolism-related genes, *insulin-like peptides* 1 (*ilp*1), *insulin-like peptides* 2 (*ilp*2), *hexamerin* 70*a* (*hex*70*a*), *hexamerin* 70*b* (*hex*70*b*), *hexamerin* 70*c* (*hex*70*c*), and *hexamerin* 110 (*hex*110); and four immunity genes, *apidaecin*, *abaecin*, *hymenoptaecin*, and *defensin-1* were investigated. *β-actin* was used as the reference gene. The primers of these genes are shown in Table 1. qPCR was performed in a 10 μL reaction volume containing 5 μL qPCR SYBR Green Master Mix (Yeasen Biotech Co., Ltd., Shanghai, China), 0.25 μL each gene-specific primer (10 μM) (Table 1), 0.5 μL cDNA template, and 4 μL RNase-free water. The reactions were performed with an ABI QuantStudio 6 Flex System (Thermo Fisher Scientific, Waltham, MA, USA). Thermal cycling conditions were 95 °C for 2 min, followed by 40 cycles of denaturation at 95 °C for 15 s, annealing at 60 °C for 65 s, and elongation at 72 °C for 20 s. The melting curve was obtained by raising the temperature from 65 to 95 °C for 10 s in 0.5 °C increments. Both technical and biological triplicates were performed in all experiments.

### 2.5. Statistical Analysis

Ct values from the upper qPCR were firstly normalized by the corresponding Ct value of the reference gene, *β-actin*, and then the relative expression levels were calculated using the 2^−△△CT^ method [39]. All statistical analyses were performed using SPSS 20.0 software (IBM, Armonk, NY, USA). All data are presented as mean ± standard error (S.E.). The significance of the differences in gene expression and the quantity of gut bacteria between the control groups and treatment groups was determined using a *t*-test. One-way analysis of variance followed by Tukey’s test (with homogeneity of variance) or Dunnett T3 test (with the heterogeneity of variance) was used for multiple comparisons of the number of gut bacteria among larvae of different ages. The significance level was set at a value of *p* < 0.05 and *p* < 0.01.

## 3. Results

### 3.1. Penicillin–Streptomycin Inhibited Symbiotic Bacteria

**Figure 1:**
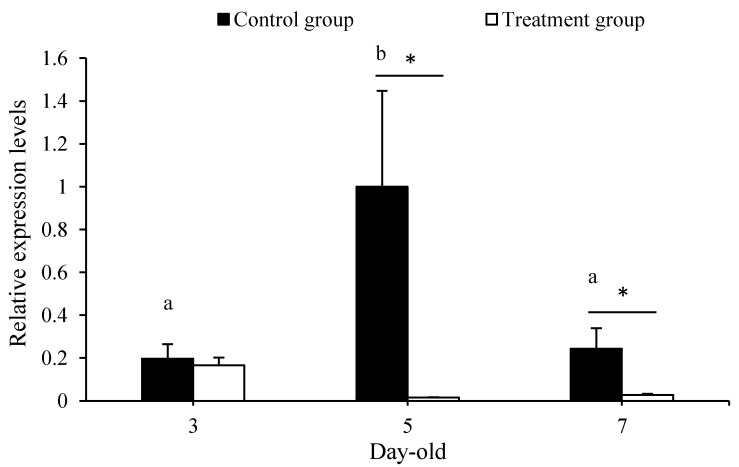
Antibiotics treatment effect on the amount of gut bacteria in larvae of different ages. The different letters on the error bars indicate the significant differences in gut bacteria amount among the 3-, 5-, and 7-day-old larvae at the 0.05 level. (Tukey’s test). The asterisk above the bars indicates a significant difference in gut bacteria amount between the treatment and corresponding control (* for *p* < 0.05).

The average expression levels of bacteria 16S rRNA in 5- and 7-day-old larvae were significantly inhibited by penicillin–streptomycin compared with the corresponding control (for 5-day-old larvae: *t* = 2.196, *p* < 0.05; for 7-day-old larvae: *t* = 2.235, *p* < 0.05), but not significantly influenced in 3-day-old larvae between the treatment groups and the control groups (*t* = 0.395, *p* > 0.05) (Figure 1). Thereby, we did not perform further analysis on the gene expression in 3-day-old larvae between the treatment groups and the control groups. Meanwhile, the amount of symbiotic bacteria in 5-day-old larvae was significantly more than that in 3- and 7-day-old larvae (*F* = 4.299, *p* < 0.05), but no significant difference was found between the 3 and 7-day-old larvae (*p* > 0.05) from the control groups.

### 3.2. Antibiotics Treatment Extended Eclosion Time and Decreased Body Weight

Based on our investigation, the pupation time of the larvae since our test established in treatment groups was 213.19 ± 0.72 h, which were statically identical to the 212.79 ± 0.90 h of larvae from the control groups (*t* = −0.35, *p* > 0.05); But the eclosion time of larvae from treatment groups was 413.69 ± 1.59 h, which were significantly longer than the 406.94 ± 1.60 h from the control group larvae (*t* = −2.966, *p* < 0.01) (Table 2). Thereby, antibiotic treatment extended the eclosion time of honeybee; furthermore, the body weights of 5-day-old larvae and newly emerged adults in the control group were 128.68 and 113.46 mg, respectively, which were also significantly higher than the 114.62 and 102.28 mg from the treated group, respectively (5-day-old larvae, *t* = 3.132, *p* < 0.01; newly emerged adults, *t* = 3.129, *p* < 0.01).

### 3.3. Antibiotics Treatment Disturbed the Expression of Development-Regulation Genes

**Figure 2:**
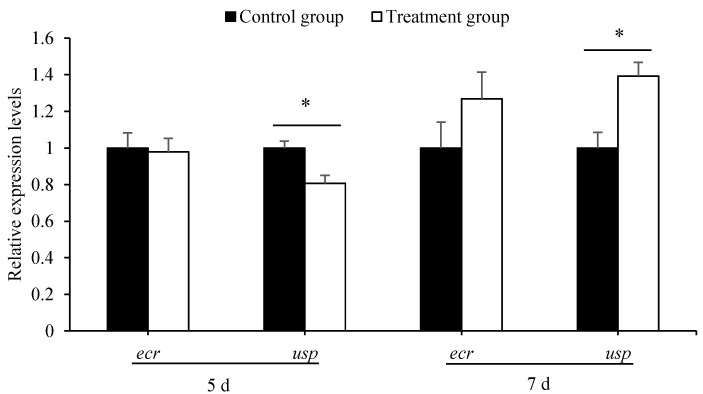
Antibiotics treatment effect on the expression of two development-regulation genes in 5- and 7-day-old *A. mellifera* larvae. The asterisk above the bars indicates a significant difference in gene expression level between the treatment and corresponding control (* for *p* < 0.05).

qPCR showed that the expression level of *usp* in 5-day-old larvae from the treatment group was significantly lower than that in larvae from the control groups (*t* = 3.359, *p* < 0.01). However, it was significantly higher in 7-day-old larvae from the treatment group than that from the control groups (*t* = −3.439, *p* < 0.01). However, the expression of *ecr*, in both 5- and 7-day-old larvae, was the same between the treatment groups and the control groups (for 5-day-old larvae, *t* = 0.201, *p* > 0.05; for 7-day-old larvae, *t* = −1.392, *p* > 0.05) (Figure 2).

### 3.4. Antibiotics Treatment Down-Regulated the Expression of Nutrient Metabolism Genes

**Figure 3:**
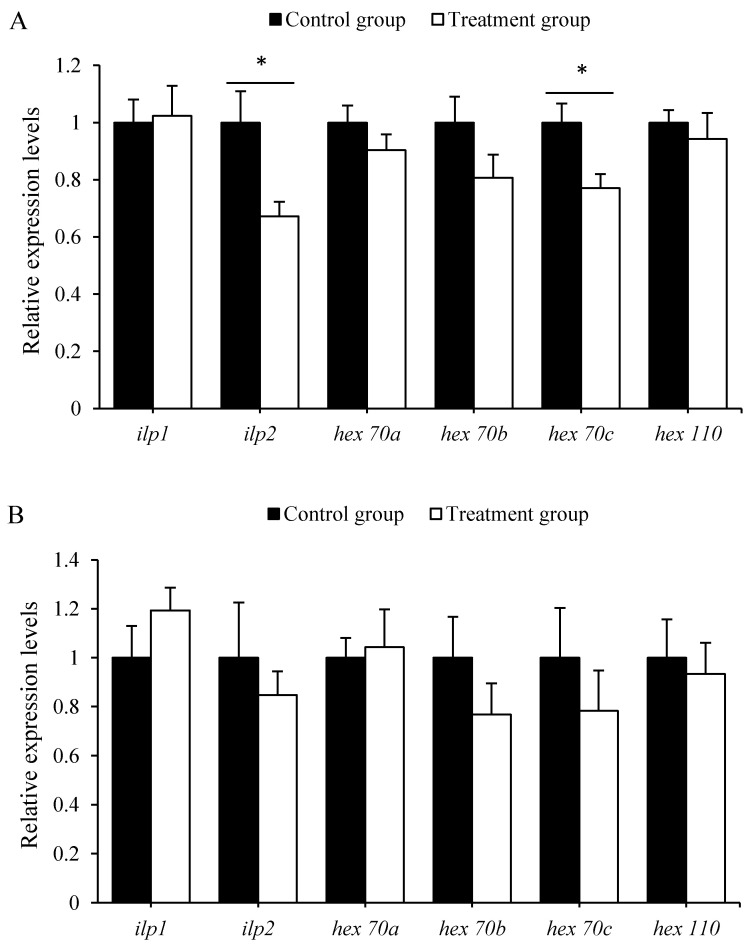
Antibiotics treatment effect on the expression of six nutrient metabolism-related genes in 5-(**A**) and 7-day-old (**B**) *A. mellifera* larvae. The asterisk above the bars indicates a significant difference in gene expression level between the treatment and corresponding control (* for *p* < 0.05).

The qRT-PCR results showed that antibiotics can significantly down-regulate the expression of *ilp*2 (*t* = 2.726, *p* < 0.01) and *hex* 70*c* (*t* = 2.776, *p* <0.01) in 5-day-old larvae (Figure 3A). However, the expression levels of *ilp*1 (*t* = −0.181, *p* > 0.05), *hex* 70*a* (*t* = 1.188, *p* > 0.05), *hex*70*b* (*t* = 1.587, *p* > 0.05), and *hex*110 (*t* = 0.571, *p* > 0.05) in 5-day-old honeybee larvae, and *ilp*1 (*t* = −1.199, *p* > 0.05), *ilp*2 (*t* = 0.623, *p* > 0.05), *hex* 70*a* (*t* = −0.248, *p* > 0.05), *hex*70*b* (*t* = 1.102, *p* > 0.05), *hex* 70*c* (*t* = 0.83, *p* > 0.05), and *hex*110 (*t* = 0.33, *p* > 0.05) in 7-day-old honeybee larvae, were not statistically different between the treated and control groups (Figure 3B).

### 3.5. Antibiotics Treatment Decreased Immunity Genes Expression

**Figure 4:**
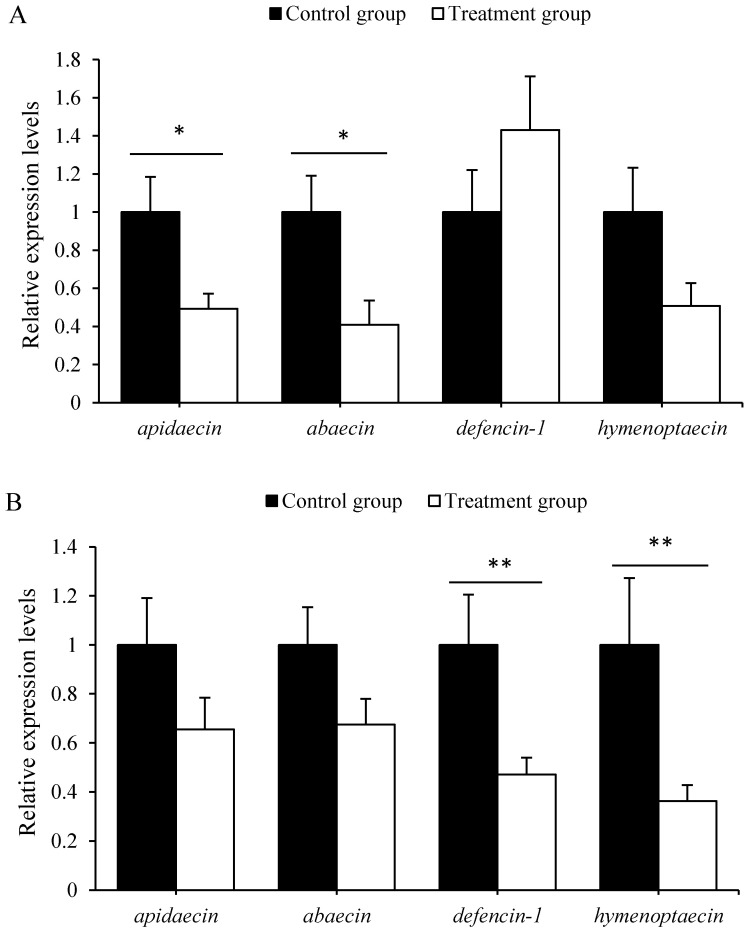
Antibiotics treatment effect on the expression of four innate immunity genes in 5- (**A**) and 7-day-old (**B**) *A. mellifera* larvae. The asterisk above the bars indicates a significant difference in gene expression level between the treatment and corresponding control (* for *p* < 0.05 and ** for *p* < 0.01).

The expression levels of *apidaecin* (*t* = 2.523, *p* < 0.05) and *abaecin* (*t* = 2.578, *p* < 0.05) in 5-day-old larvae, and *defencin*-1 (*t* = 2.444, *p* < 0.05) and *hymenoptaecin* (*t* = 2.278, *p* < 0.05) in 7-day-old larvae, were significantly decreased in treated groups (Figure 4). Therefby, antibiotics treatment down-regulated the expression of the immune genes in honeybee larvae.

## 4. Discussion

Many studies involving gut microbiota have provided a deeper understanding of the ecological and functional dynamics of gut environments and their benefit in the health of host honeybees [19,25,28,40,41]. Compared with the microbiota in mammals, the gut microbiota in honeybees is more simple and host-specific [19,42]. In recent years, the adult honeybee of *A. mellifera* has increased as a valuable model system for researching the relationship between its gut microbiota and the host [43]. However, the microbiota in honeybee larvae (primarily the *Acetobacteraceae* (*Alpha2.2*) and *Lactobacillus*) is generally considered less important due to its absence of host specificity and it being easily affected by the food [19,24]. Previous reports have shown that the microbiota in honeybee larvae also play a positive role in nutrient uptake and defense against pathogens [25,41,44]. In this study, to determine the effect of gut microbiota on the whole fitness of honeybee larvae, we disrupted the gut bacteria by using antibiotics and found that the treated larvae showed a lower fitness in development, nutrient metabolism, and immune competence than the control larvae.

Gut microbiota can supply amino acids, proteins and vitamins, and other essential materials, and regulate some pathways for host growth, development, and metabolism [45,46]. This result is consistent with previous reports that antibiotics cause rapid and profound alterations of gut microbiota [40,47]. Meanwhile, the decrease in gut microbiota leads to a reduction in nutrient metabolism and body weight, and extended the growth time of larvae. The body weight of honeybee larvae was determined by the nutrient intake and related metabolism. Our investigation showed that the body weights of 5-day-old larvae and newly emerged adults from the antibiotics-treated larvae were both significantly lower than the control. Moreover, the expression levels of nutrient metabolism-related genes, such as *ilp2*, *hex 70a*, *hex 70b*, *hex70c*, and *hex 110*, in 5- and 7-day-old antibiotics-treated larvae were lower than those of the controls. Nutrient-sensitive insulin-like peptides (ILPs) have profound effects on invertebrate metabolism, nutrient storage, fertility, and aging [48]. Hexamerins are the most abundant proteins in larval hemolymph and serve as storage proteins for gonad development, egg production, and foraging activity [49]. The expression levels of both ILPs and hexamerins reflected the nutritional status of larvae [50,51]. Our data indicate that the disruption of gut bacteria by antibiotics could decrease the expression of nutrient metabolism-related genes, which in turn, decreased the body weights of 5-day-old larvae and newly emerged adults. Thereby, gut microbiota in larvae could to some extent determine the host larvae’s nutrition state.

As a complete metamorphosis insect, the development rhythm was coordinately regulated by the juvenile hormone (JH) and molting hormone (20-hydroxyecdysone, 20E) [52,53]. The ecdysteroid receptor (EcR) and ultraspiracle protein (USP) were the key genes responsible for the transduction of the JH/20E signals during metamorphosis development. Our results showed that the antibiotics-treated larvae have significantly longer developmental periods than the control larvae, and the expression levels of *usp* in 5- and 7-day-old antibiotics-treated larvae were altered, which might disturb the normal development process leading to the prolonged development period of adult honeybees (Table 2). Such extension in the developmental periods of treated larvae might come from either the decreased hexamerins for building the pupae tissues or some metabolites for the synthesis of JH/20E by gut microbiota, which were already disrupted by antibiotics [49].

Successful immunity is indispensable for the survivorship of all organisms. Like other insects, honeybees have evolved innate immunity through synthesizing a variety of antimicrobial peptides (AMPs) by their fat bodies to defend against microbial infection [54], which are regulated by Toll, Imd, JNK, and JAK/STAT signaling pathways [47,55,56]. The vital immune regulating roles of gut bacteria have been well reported in adult honeybees [47]. We found here that the expression levels of *apidaecin*, *abaecin* in 5-day-old larvae and *defensing*-1, *hymenoptaecin* in 7-day-old larvae were significantly down-regulated in antibiotics-treated larvae (Figure 4). Therefore, gut bacteria in honeybee larvae, despite their lower host specificity, were essential in regulating larval immune response, which was similar to what happened in adult honeybees. Such a result verified that the antibiotics could cause a huge negative impact on larva bees as well. In practice, antibiotics were also used to control honeybee larval diseases, such as American Foulbrood Diseases (AFB) and European Foulbrood Diseases (EFB), caused by pathogenic bacteria [48]. However, the benefit of the control of the disease might not counteract the loss caused by using the antibiotic. The beekeeper should avoid using antibiotics for honeybee disease control.

## 5. Conclusions

Despite their variability and low host specificity, the gut microbiota in honeybee larvae could affect the host’s nutrient metabolism, increase the larval weight, and participate in the developmental regulation and immune response, which were similar to the roles of the gut microbiota played in adult bees. They increased the fitness of host larvae and were, therefore, indispensable for the development of honeybee larvae. The disruption of gut microbiota by antibiotics could decrease the nutrient metabolism, decrease the body weight, postpone the development, and lower the immunity of larvae. These results fulfill the biological functions of gut microbiota in honeybee larvae and provide fundamental information for future research and application in this field.

## Figures and Tables

**Table 1:** Primers used for quantitative PCR.

Genes		Primer Sequences (5′-3′)	Gene ID	Reference
Reference gene	*β-actin*	F: TTGTATGCCAACACTGTCCTTT R: TGGCGCGATGATCTTAATTT	NM_001185145.1	Simone et al. [33]
16S rRNA gene	*unibac*	F: AGAGTTTGATCCTGGCTCAG R: CTGCTGCCTCCCGTAGGAGT		Schwarz et al. [34]
Development-related genes	*ecr*	F: TGCCAACACTGTCCTTTCTG R: AAAGAGCCAGGCTGCGACAA	XM_016913298.2	Liu et al. [35] Liu et al. [35]
	*usp*	F: GCCAAGATGATGAAGAAGGAGA R: GGTTGATGAGGCTTGCTGTGTC	NM_001011634.2	
Nutrient metabolism-related genes	*ilp*1	F: CGATAGTCCTGGTCGGTTTG R: CAAGCTGAGCATAGCTGCAC	XM_026442143.1	De Azevedo et al. [36]
	*ilp*2	F: TTCCAGAAATGGAGATGGATG R: TAGGAGCGCAACTCCTCTGT	NM_001177903.1	
	*hex* 70*a*	F: GCCTTCAGTTTGGTCGGTGC R: GCTGGTTGAGCGACGCGATA	NM_001110764.1	Zhang [37]
	*hex* 70*b*	F: AACAGCCACGAATCCGTCTT R: CAGGCTTGTCCAGAGGGAAG	NM_001011600.1	Zheng et al. [3] Zheng et al. [3] Zheng et al. [3]
	*hex* 70*c*	F: TAAGGCAGGCAGACTTGAGC R: AAACGCTGTGGTGAACATGC	NM_001098717.1	
	*hex* 110	F: ACGGACAATACCCGAACACC R: AGCATGCTGATGCCTCTGTT	NM_001101023.1	
Innate virus immunity genes	*abaecin*	F: CAGCATTCGCATACGTACCA R: GACCAGGAAACGTTGGAAAC	NM_001011617.1	Simone et al. [33] Evans et al. [38] Evans et al. [38]
	*hymenoptaecin*	F: CTCTTCTGTGCCGTTGCATA R: GCGTCTCCTGTCATTCCATT	NM_001011615.1	
	*defencin*-1	F: TGCGCTGCTAACTGTCTCAG R: AATGGCACTTAACCGAAACG	NM_001011616.2	
	*apidaecin*	F: TTTTGCCTTAGCAATTCTTGTTG R: GTAGGTCGAGTAGGCGGATCT	NM_001011613.1	Evans et al. [38]

**Table 2:** Effect of antibiotics on development period and body weight of honeybee larvae.

Treatment	Development Period (h)		Body Weight (mg)	
	Pupation	Eclosion	5-Day-Old Larvae	Newly Emerged Adults
Control group	212.79 ± 0.90	406.94 ± 1.60	128.68 ± 2.95	113.46 ± 2.69
Treatment group	213.19 ± 0.72	413.69 ± 1.59 **	114.62 ± 3.39 **	102.38 ± 2.30 **

The asterisk above the bars indicates a significant difference in development period and body weight between the treatment and corresponding control (** for *p* < 0.01).

## Data Availability

Not applicable.
